# Estimating Natural Mortality of Atlantic Bluefin Tuna Using Acoustic Telemetry

**DOI:** 10.1038/s41598-019-40065-z

**Published:** 2019-03-20

**Authors:** Barbara A. Block, Rebecca Whitlock, Robert J Schallert, Steve Wilson, Michael J. W. Stokesbury, Mike Castleton, Andre Boustany

**Affiliations:** 1 Hopkins Marine Station, Stanford University, Pacific Grove, California, 93950 USA; 2 Tuna Research and Conservation Center, Stanford University, Hopkins Marine Station, Pacific Grove, California, 93950 USA; 3 Swedish University of Agricultural Sciences, Department of Aquatic Resources, Institute of Freshwater Research, Stångholmsvägen 2, SE-178 93, Drottningholm, Sweden; 4 Biology Department, Acadia University, Wolfville, NS B4P 2R6 Canada; 5 Marine Geospatial Ecology Lab, Nicholas School of the Environment, Duke University, Durham, NC 27708 USA; 6 Monterey Bay Aquarium, 886 Cannery Row, Monterey, CA 93940 USA

## Abstract

Atlantic bluefin tuna (*Thunnus thynnus*) are highly migratory fish with a contemporary range spanning the North Atlantic Ocean. Bluefin tuna populations have undergone severe decline and the status of the fish within each population remains uncertain. Improved biological knowledge, particularly of natural mortality and rates of mixing of the western (GOM) and eastern (Mediterranean) populations, is key to resolving the current status of the Atlantic bluefin tuna. We evaluated the potential for acoustic tags to yield empirical estimates of mortality and migration rates for long-lived, highly migratory species such as Atlantic bluefin tuna. Bluefin tuna tagged in the Gulf of St. Lawrence (GSL) foraging ground (2009–2016) exhibited high detection rates post release, with 91% crossing receiver lines one year post tagging, 61% detected after year two at large, with detections up to ~1700 days post deployment. Acoustic detections per individual fish ranged from 3 to 4759 receptions. A spatially-structured Bayesian mark recapture model was applied to the acoustic detection data for Atlantic bluefin tuna electronically tagged in the GSL to estimate the rate of instantaneous annual natural mortality. We report a median estimate of 0.10 yr^−1^ for this experiment. Our results demonstrate that acoustic tags can provide vital fisheries independent estimates for life history parameters critical for improving stock assessment models.

## Introduction

Atlantic bluefin tuna *Thunnus thynnus*, is distributed throughout the North Atlantic Ocean and exploited by fisheries throughout its range. Conventional tagging^1,2^, electronic tagging^3–7^, genetics^8–10^, organochlorine tracer analysis^11^, and otolith microchemistry studies^12–14^ indicate the existence of two separate spawning populations with origins in the Mediterranean Sea and Gulf of Mexico. Additional spawning populations may exist however Atlantic bluefin tuna are currently managed by the International Commission for the Conservation of Atlantic Tunas (ICCAT) as two stocks (western and eastern) separated by the 45°W meridian although extensive mixing on foraging grounds is known to occur^3,12^. Both stocks are considered to be in rebuilding phases. Current total allowable catches (TACs) are 28,200 tonnes in the eastern Atlantic and 2,350 tonnes in the west Atlantic^15^. Significant increases in TACs are projected in the next few years, particularly in the eastern Atlantic and Mediterranean Sea where quotas are on target for 36,000 tonnes by 2020. The western Atlantic bluefin tuna populations declined in the 1960s to 1970s to a low spawning stock biomass that has since remained stable through implementation and enforcement of stringent catch quotas^15,16^. In the US, domestic management is focused on preventing overfishing of the quota, proper allocation to sectors of the fishery, protection on the Gulf of Mexico spawning grounds, and rebuilding of the western population under the 2006 Consolidated Highly Migratory Species Fishery Management Plan.

Stock assessment models rely on a realistic description of species’ biology and ecology to yield unbiased estimates of stock status. Estimates of the current spawning stock biomass (SSB) and its ratio to the historical SSB (depletion) are central to ICCAT’s stock assessment process and the provision of management advice^16^. Estimates of current SSB and depletion in turn can depend on assumptions made about productivity, movements and stock mixing, among other things^17–21^. One of the most important determinants of stock productivity is the rate of natural mortality (*M*), which has traditionally been difficult to estimate^22^. Natural mortality has been estimated for Atlantic bluefin tuna using electronic tagging data^23^, although values of age-specific *M* for western Atlantic bluefin tuna are still considered uncertain by ICCAT. In 2017, the western assessment used an age-varying rate derived from the Lorenzen method^23^ scaled to *M* = 0.10 at ages 14–16+, while the eastern assessment used a Lorenzen curve scaled to *M* = 0.10 at ages 20+ ^24^. A sensitivity analysis found that a lower rate of terminal *M* for the western stock is associated with lower estimates of recruitment and SSB.

The degree of mixing between eastern and western stocks is a further key uncertainty in the Atlantic bluefin tuna assessment. ICCAT currently uses separate assessment models for eastern and western Atlantic bluefin tuna (i.e. population mixing is not accounted for). Estimates of population-specific depletion can be biased when catch removals are not attributed to the correct stock of origin^18–20^. An analysis using simulated data has indicated that accouting for mixing is particularly important for the western stock to obtain unbiased estimates of absolute stock size^25^. Information about the movement and relative abundance of different populations in time and space is key to correctly quantifying the contributions of those populations to fishery catches in mixing models. Electronic tagging of Atlantic bluefin tuna has emerged as a powerful tool for learning about many aspects of the biology and ecology of bluefin tunas^26–35^. Data obtained from electronic tags has the potential to improve estimates of key model parameters, such as rates of fishing and natural mortality, and migration and mixing. Conventional and electronic tagging have revealed the details of large-scale migrations of juvenile, adolescent and mature bluefin tuna, the understanding of which is central to the proper management of this species^3–7,17–20,26–33^. Information from electronic tagging and biological markers that provide origin of the tagged fish, allows estimation of population-specific movement patterns. Tagging, otoliths and genetics indicate that the amount of trans-Atlantic crossing varies depending upon population of origin, year examined, age of catch, and possibly sex. Tagging studies have indicated higher rates of trans-oceanic movements of eastern origin fish to the western Atlantic than vice versa, likely due to the much larger size of the eastern population^5^ and the more limited distribution of the GOM population. Together with genetic markers, results from electronic tagging can also be informative about catch composition, demonstrating for example that western Atlantic bluefin tuna fisheries target mixed populations along the eastern seaboard of North America^5,18^.

Using this new knowledge from electronic tagging data to build biologically plausible models is vital to minimizing bias in assessments of stock status. Despite the rapid advances in our understanding of Atlantic bluefin tuna biology, key questions remain about population mixing, productivity, recruitment dynamics, maturity schedules, abundance trends and the number and stock origin of fish harvested by western Atlantic fisheries. To date, almost all electronic tagging of Atlantic bluefin tuna has been focused on deployments of archival and pop-up satellite archival tags. Acoustic tags have the potential to provide valuable information about the biology of Atlantic bluefin tuna, given their high detection rate, their longevity, and the independence of detections from fishing activity.

In this paper, we examine the use of acoustic tags in combination with Ocean Tracking Network (OTN)- deployed acoustic receiver lines across entrances of the Gulf of St. Lawrence to: a) examine timing of arrival and departures of Atlantic bluefin tuna foraging in the GSL, b) determine the fidelity to foraging grounds annually to estimate how many fish return to the GSL, c) estimate survivorship using a multistate Bayesian mark-recapture model, and d) test if fish acoustically tagged and released in North Carolina waters recruit into the GSL. The GSL may serve as a unique location for long term monitoring of the Atlantic bluefin tuna fishery, due to the extraordinary investments Canada has made in the OTN infrastructure in this region. Strategic underwater receiver lines are now in place in many location of Canadian coastal waters and opportunistic investments have placed additional receivers along the US coastline from Maine to Florida. Conventional tags placed simultaneously on fish tagged with the acoustic tags provide an additional set of long-term marks necessary to generate estimates of natural and fishing mortality similar to previous studies conducted on Atlantic and Pacific bluefin tuna (*Thunnus Orientalis*) using archival and pop-up satellite tags^23,35^.

## Methods

From 2009–2016, 128 Atlantic bluefin tuna were electronically tagged and released with V16-4H, Vemco acoustic tags in the Gulf of St. Lawrence, Canada and in the waters off North Carolina, USA. Below, we develop a model for the 101 acoustic tagged Atlantic bluefin tuna released during 2009–2015 (Table 1). Fish were caught on commercial Atlantic bluefin tuna fishing vessels, permitted to conduct scientific tagging, in the fall months off Port Hood on Cape Breton Island, Nova Scotia. The fish were all caught on rod and reel with live or freshly caught dead Atlantic mackerel (*Scomber scombrus*) or Atlantic herring (*Clupea harengus*) bait. During the tagging campaigns, one vessel was designated a tagging boat and multiple fishing vessels caught bluefin tuna on rod and reel. The fish were “transferred” to the designated tagging boat, the F/V Bay Queen IV, which had a large deck and transom. All bluefin tuna were brought on board the vessels using methodologies described previously^3,5,17^. In addition to these Canada deployments, four fish were caught on trolling lures in North Carolina waters in March 2013 using sport fishing vessel, tagged and released^4^.

**Table 1:** Atlantic bluefin tuna acoustic tag deployments measured length (CFL), tagging date, and detection history.

Topp ID	Code	CFL	Tagging Date	Lat	Long	First Detect	Last Detect	# Detects	In model
5109023	60050	250	10/18/2009	46.11	−61.74	7/12/2010	11/4/2011	168	yes
5109024	60051	273	10/18/2009	46.14	−61.63	6/15/2010	9/15/2011	167	yes
5109026	60056	269	10/22/2009	46.21	−61.55	6/20/2010	9/12/2014	141	yes
5109027	60057	293	10/22/2009	46.18	−61.56	6/22/2010	12/19/2011	250	yes
5109028	60058	233	10/22/2009	46.2	−61.6			0	yes
5109029	60060	277	10/24/2009	46.21	−61.61	6/21/2010	10/21/2011	100	yes
5109030	60047	261	10/24/2009	46.24	−61.61	6/30/2010	9/23/2012	175	yes
5109031	60052	268	10/30/2009	46.34	−61.57	6/15/2010	11/16/2011	651	yes
5109032	60049	262	10/30/2009	46.34	−61.57			0	yes
5110066	60053	272	9/24/2010	46.06	−62.1			0	yes
5110067	60054	293	9/24/2010	46.05	−62.1	9/28/2010	10/21/2011	78	yes
5110091	45851	197	10/16/2010	46.3	−61.4	10/17/2010	1/8/2013	149	yes
5110092	45849	194	10/16/2010	46.25	−61.36	10/18/2010	10/18/2010	8	yes
5111014	19214	247	9/23/2011	46.04	−61.6	10/7/2011	10/22/2013	220	yes
5111018	19348	219	9/24/2011	46.04	−61.61	9/28/2011	11/8/2012	191	yes
5111019	19351	237	9/24/2011	46.04	−61.6			0	yes
5111020	19215	246	9/24/2011	46.03	−61.62	10/13/2011	11/9/2011	172	yes
5111021	19216	299	9/24/2011	46.04	−61.62	9/27/2011	9/27/2011	7	yes
5111029	19349	175	9/29/2011	46.01	−61.71	2/1/2012	11/9/2015	749	yes
5111030	45847	252	9/29/2011	46.03	−61.72	10/11/2011	10/11/2011	3	yes
5111035	19347	244	10/3/2011	46.07	−61.74	10/6/2011	11/17/2012	20	yes
5111036	19350	203	10/3/2011	46.08	−61.7	10/6/2011	12/11/2013	421	yes
5111037	19219	243	10/3/2011	46.06	−61.66	10/7/2011	11/12/2012	206	yes
5111038	19221	211	10/3/2011	46.06	−61.67	10/6/2011	11/17/2013	549	yes
5111039	45845	239	10/3/2011	46.07	−61.7	10/6/2011	12/15/2013	166	yes
5111040	45850	210	10/3/2011	46.06	−61.68	10/7/2011	10/19/2013	112	yes
5111043	46020	220	10/13/2011	46.18	−61.46	10/27/2011	10/24/2013	514	yes
5111044	46021	199	10/14/2011	46.18	−61.52	10/16/2011	10/5/2012	128	yes
5111047	45844	246	10/19/2011	46.09	−61.55	10/28/2011	10/19/2013	180	yes
5111048	19217	269	10/21/2011	46.1	−61.57	10/27/2011	11/8/2012	104	yes
5111049	19218	209	10/21/2011	46.1	−61.56	10/25/2011	11/12/2013	287	yes
5111054	45848	221	10/23/2011	46.01	−61.7			0	yes
5111057	45846	193	10/25/2011	45.97	−61.72	10/27/2011	10/27/2011	3	yes
5111058	19220	215	10/16/2011	46.08	−61.58	10/19/2011	10/23/2013	371	yes
5112028	33775	270	9/23/2012	46.02	−62.2	9/27/2012	10/15/2014	145	yes
5112029	33777	235	9/24/2012	46.02	−62.22	10/12/2012	10/21/2013	246	yes
5112030	33776	283	9/24/2012	46.01	−62.23	9/28/2012	10/19/2013	57	yes
5112031	33778	222	9/24/2012	46.01	−62.32	10/18/2012	11/17/2013	342	yes
5112032	33779	260	9/24/2012	46.01	−62.31	10/1/2012	7/23/2013	34	yes
5112033	33780	278	9/24/2012	46.01	−62.31	10/19/2012	11/5/2014	669	yes
5112034	33781	270	9/29/2012	46	−62.33	10/5/2012	7/22/2014	82	yes
5112035	33783	259	9/29/2012	46	−62.33	10/6/2012	3/1/2014	196	yes
5112036	33785	261	9/29/2012	46	−62.33	10/6/2012	10/15/2013	133	yes
5112037	33786	268	9/29/2012	46.04	−62.31	10/14/2012	10/30/2014	432	yes
5112038	33784	277	10/5/2012	46	−62.31	11/1/2012	9/19/2013	297	yes
5112039	33787	273	10/5/2012	45.98	−62.35	10/8/2012	7/20/2014	240	yes
5112040	33788	218	10/5/2012	46	−62.34	10/15/2012	11/5/2013	412	yes
5112041	33789	284	10/5/2012	46	−62.34	10/27/2012	10/20/2014	4759	yes
5112042	33790	282	10/5/2012	46	−62.37	10/29/2012	1/11/2013	102	yes
5112043	19222	259	10/8/2012	46.01	−62.22	10/11/2012	9/8/2014	294	yes
5112044	19225	265	10/9/2012	46.11	−61.98	10/24/2012	11/6/2012	60	yes
5112045	33145	271	10/9/2012	46.11	−61.98	10/11/2012	10/21/2014	182	yes
5112046	19227	250	10/9/2012	46.09	−62.01	10/25/2012	11/16/2014	117	yes
5112047	19224	225	10/9/2012	46.09	−61.99			0	yes
5112048	33792	221	10/9/2012	46.1	−62.01	10/18/2012	11/2/2014	861	yes
5113001	33731	180	3/23/2013	35.53	−74.83	8/27/2013	8/23/2015	228	yes
5113002	33146	189	3/23/2013	35.38	−74.93			0	yes
5113003	33150	183	3/23/2013	35.37	−74.87	6/30/2013	10/20/2015	855	yes
5113004	33144	174	3/30/2013	35.02	−75.13	6/11/2013	7/2/2014	74	yes
5113014	33143	272	9/28/2013	45.98	−61.61	10/24/2013	12/14/2013	63	yes
5113015	33149	284	9/28/2013	45.99	−61.61	9/30/2013	10/21/2013	106	yes
5113016	33151	251	9/28/2013	45.99	−61.61	7/5/2014	8/15/2015	102	yes
5113017	26303	282	9/29/2013	45.99	−61.61	10/3/2013	6/16/2014	229	yes
5113018	26304	266	9/29/2013	45.98	−61.61			0	
5113019	26305	262	9/29/2013	45.97	−61.61	10/26/2013	4/26/2015	33	yes
5113020	26277	294	9/29/2013	45.96	−61.6	10/17/2013	10/30/2013	34	yes
5113021	26281	265	9/29/2013	45.98	−61.61	10/14/2013	7/22/2017	453	yes
5113022	26306	271	9/29/2013	45.97	−61.62	10/16/2013	10/7/2016	594	yes
5113023	26278	271	9/30/2013	45.99	−61.62	10/9/2013	4/15/2015	779	yes
5113024	26280	274	9/30/2013	45.98	−61.62	10/16/2013	1/28/2014	328	yes
5113025	26307	269	9/30/2013	45.97	−61.62	10/3/2013	10/6/2017	866	yes
5113026	26279	246	9/30/2013	45.98	−61.61	10/4/2013	4/28/2017	1703	yes
5113027	26282	296	9/30/2013	45.98	−61.62	10/28/2013	10/28/2014	498	yes
5113028	33152	239	9/30/2013	45.98	−61.62	10/4/2013	10/21/2014	219	yes
5113029	33157	277	9/30/2013	45.97	−61.62	10/26/2013	5/22/2014	121	yes
5113030	33155	267	9/30/2013	45.97	−61.63	10/6/2013	11/2/2013	106	yes
5113031	33730	269	10/1/2013	45.97	−61.63	10/13/2013	8/10/2016	113	yes
5113032	33154	313	10/1/2013	45.97	−61.62			0	yes
5113033	33156	298	10/1/2013	45.96	−61.63	10/5/2013	12/2/2014	761	yes
5113034	33732	276	10/2/2013	45.97	−61.62	10/5/2013	10/5/2013	12	yes
5113035	33148	282	10/2/2013	45.98	−61.62	10/4/2013	10/3/2014	188	yes
5113036	33153	241	10/2/2013	45.98	−61.61	10/5/2013	9/29/2015	779	yes
5113037	33737	297	10/2/2013	45.98	−61.62	10/4/2013	11/6/2015	218	yes
5114009	27167	250	10/18/2014	46.06	−61.59	10/20/2014	8/28/2017	574	yes
5114010	27168	229	10/18/2014	46.07	−61.58	10/24/2014	7/11/2017	3274	yes
5114011	27169	239	10/19/2014	46.03	−61.59	10/30/2014	12/7/2017	208	yes
5114012	26929	250	10/19/2014	46	−61.62	10/29/2014	8/28/2016	70	yes
5114013	26931	230	10/19/2014	46.03	−61.61	10/21/2014	10/21/2017	140	yes
5114014	26928	251	10/19/2014	46.03	−61.61	10/22/2014	11/22/2015	76	yes
5114015	26933	231	10/21/2014	46.01	−61.61	10/28/2014	7/6/2017	116	yes
5114016	26935	265	10/21/2014	46.02	−61.61	10/25/2014	10/24/2016	169	yes
5114017	26940	260	10/21/2014	46.02	−61.61	11/5/2014	2/4/2017	296	yes
5114018	65166	258	10/21/2014	46.02	−61.61	10/23/2014	11/10/2017	378	yes
5114019	26938	226	10/21/2014	46.02	−61.62	10/24/2014	12/12/2015	124	yes
5114020	33147	247	10/22/2014	46.1	−61.55	10/24/2014	11/5/2014	80	yes
5114021	33735	237	10/22/2014	46.1	−61.56	10/25/2014	3/30/2017	259	yes
5114022	19228	252	10/22/2014	46.13	−61.53	10/29/2014	10/12/2016	57	yes
5114023	65167	272	10/22/2014	46.14	−61.53	10/24/2014	10/24/2014	6	yes
5114024	13836	270	10/22/2014	46.15	−61.49	10/25/2014	9/30/2016	64	yes
5114026	13838	226	10/26/2014	46.14	−61.49	11/4/2014	11/4/2014	4	yes
5115001	20982	250	10/22/2015	46.18	−61.51	10/23/2015	10/24/2015	13	yes
5115002	20983	229	10/22/2015	46.05	−61.65	10/29/2015	1/11/2018	168	yes
5116001	20987	233	8/25/2016	46.3	−62.55	10/3/2016	10/3/2016	14	
5116002	20992	235	8/26/2016	46.15	−62.62			0	
5116003	20985	227	8/31/2016	46.37	−61.5			0	
5116004	20984	241	9/23/2016	45.99	−61.61	10/4/2016	10/5/2016	5	
5116005	20999	241	9/23/2016	45.99	−61.62	9/25/2016	11/5/2017	82	
5116006	21001	251	9/24/2016	45.98	−61.61			0	
5116007	20997	255	9/24/2016	45.96	−61.63	9/27/2016	2/10/2018	338	
5116008	21000	245	9/24/2016	45.97	−61.62	10/16/2016	10/19/2017	84	
5116009	20994	252	9/24/2016	45.97	−61.63	10/12/2016	8/18/2017	48	
5116010	20995	264	9/24/2016	45.98	−61.62	9/27/2016	11/12/2017	76	
5116011	20996	243	9/28/2016	45.99	−61.63	10/15/2016	10/15/2016	3	
5116012	20991	251	9/28/2016	45.99	−61.62	10/15/2016	7/7/2017	27	
5116013	20988	219	9/28/2016	45.99	−61.61	10/12/2016	10/15/2017	42	
5116014	20998	244	9/28/2016	45.99	−61.62	10/2/2016	11/14/2016	75	
5116015	20993	229	9/28/2016	45.98	−61.62	10/4/2016	6/30/2017	73	
5116016	20990	253	10/1/2016	46.07	−61.59	10/16/2016	11/11/2016	61	
5116017	20986	233	10/1/2016	46.03	−61.68	10/16/2016	7/31/2017	52	
5116018	20989	224	10/1/2016	46.07	−61.66	10/12/2016	7/6/2017	46	
5116019	59948	255	10/1/2016	46.07	−61.66	8/18/2017	8/18/2017	11	
5116020	59938	249	10/1/2016	46.06	−61.76	10/3/2016	8/23/2017	87	
5116021	54916	182	10/1/2016	46.05	−61.75	11/4/2016	11/17/2016	51	
5116022	59945	211	10/1/2016	46.05	−61.75	10/5/2016	12/14/2017	44	
5116023	59947	234	10/2/2016	46.04	−61.81	10/20/2016	8/8/2017	26	
5116025	59944	281	10/2/2016	46.05	−61.85	10/13/2016	10/17/2016	67	
5116026	54928	228	10/2/2016	46.08	−61.88	10/5/2016	8/19/2017	34	
5116028	59949	267	10/4/2016	46.12	−61.52	5/23/2017	6/30/2017	15	

**Figure 1:**
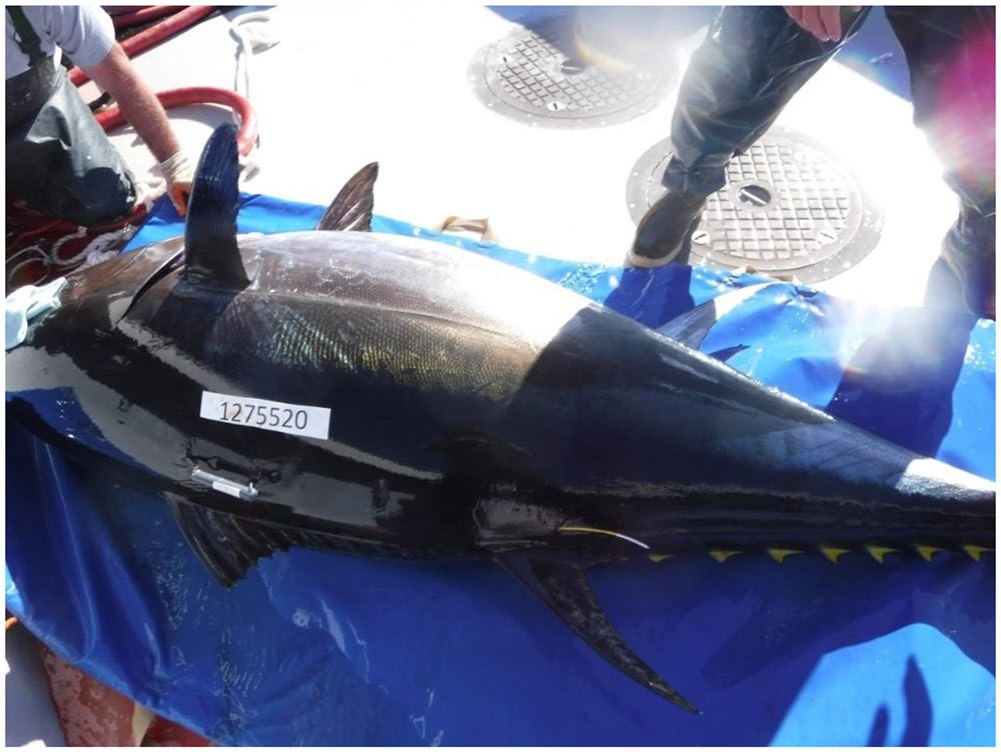
External acoustic tag attachment for an Atlantic bluefin tuna with two titanium darts in the dorsal musculature.

Once a bluefin tuna was caught by rod and reel, the fish was reeled in and leadered to the open transom door. By placing a titanium or stainless steel lip hook, carefully behind the lower jawbone, we are able to pull the fish through the transom door and onto a wet vinyl mat. A saltwater hose was inserted in the mouth to oxygenate the tuna gills while on deck and a soft cloth soaked in a fish protectant solution (PolyAqua®) was placed over the eyes to keep the fish calm^3,33^. Curved fork length (CFL) of the fish was measured to the nearest mm with a flexible tape measure, fish were also sampled for fin clips for genetics, tagged and released. When possible pictures of the electronic tag positions were obtained upon release (Fig. 1). All electronic tagging procedures with the Atlantic Bluefin tuna were conducted under protocols approved by the Stanford University Administrative Panel on Laboratory Care in accordance with the Institutional Animal Care and Use Committee’s proper guidelines and Acadia Animal Care Committee protocol #18-11. In addition, all procedures were approved under permits issued by Fisheries and Oceans Canada license # SG-RHQ-18-159A.

For this experiment all Vemco acoustic tags were packaged in a plastic “shark case” with 5 mm holes drilled in at both ends of the tag (Fig. 1). The holes were enlarged for the attachment leaders by hand boring with a file or dremel tool, and carefully smoothed to prevent any interaction of the edges of the hole with the materials used to construct the leaders. The tags were secured to the fish externally using a two-point attachment technique, with a custom titanium dart on each end of the acoustic tag. Tags were inserted into the dorsal musculature of the fish at depths of 15.2 to 17.8 cm depending upon the size of the bluefin tuna. The materials in the leader consisted of a single layer of 180 kg monofilament (Moi Moi Hard), a cover layer of aramid braided cord that provided increased abrasion resistance over the monofilament, and up to two layers of heat shrink wrap. Pop-up satellite archival tags (Wildlife Computers MK-10 and mini-PATs) were attached to a subset of the acoustically tagged tuna and tracks from these satellite tags were reported on previously^17,33^. Information from these pop-up satellite archival tags are not used in the present model and analysis.

**Figure 2:**
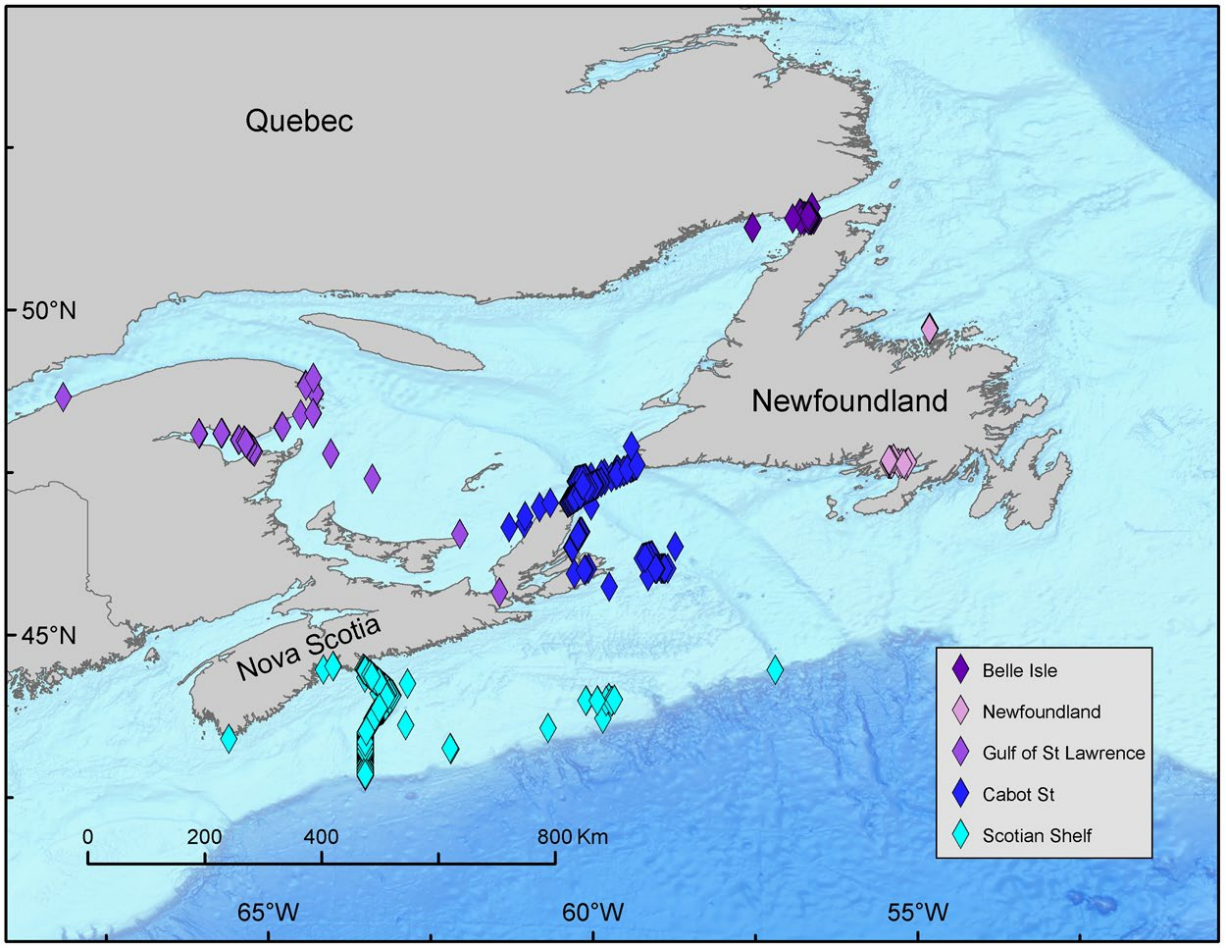
Locations of acoustic detections in Canadian waters including Vemco receivers on moorings, and small detectors on electronically tagged Grey seals and wave gliders. This map was generated in ESRI ArcMap software (Version:10.3.1 & http://desktop.arcgis.com/en/arcmap/10.3/main/get-started/whats-new-in-arcgis-1031.htm).

**Figure 3:**
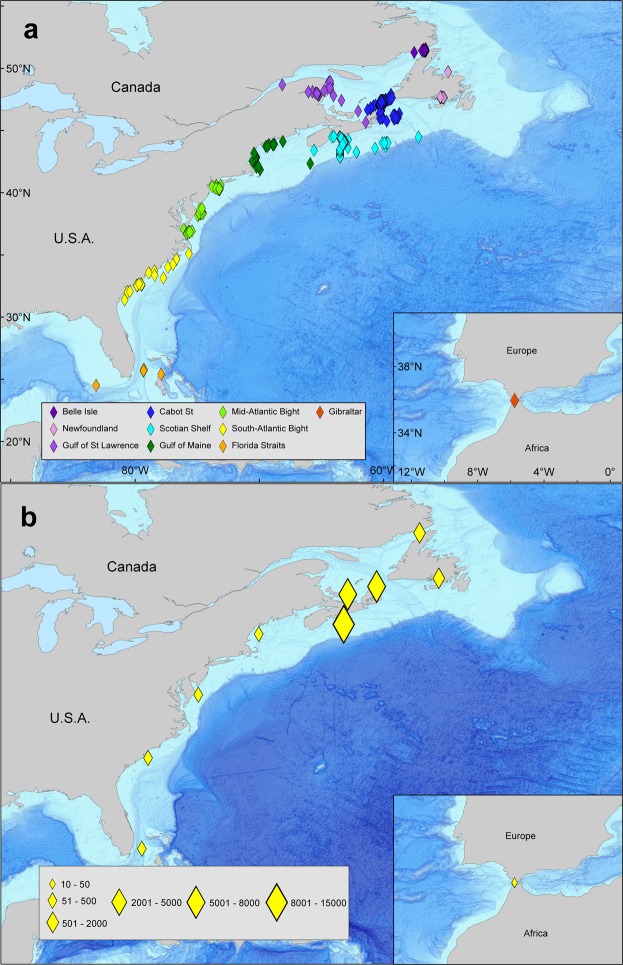
Receiver locations of detected Atlantic bluefin tuna. (**a**) All receiver locations. (**b**) Graduated symbol detection count by region. This map was generated in ESRI ArcMap software (Version:10.3.1 & http://desktop.arcgis.com/en/arcmap/10.3/main/get-started/whats-new-in-arcgis-1031.htm).

Acoustic receiver lines using VR4 UMs receivers were deployed and maintained by OTN. They were initially placed across a portion of the Cabot Strait and across the Scotian shelf off Halifax, Canada in the summer of 2007 (Fig. 2). The receiver array used to enclose the GSL was partially installed when the project was initiated. This line was completed in late 2008 and spanned the entire Cabot Strait and the Strait of Belle Isle, which together provides an electronic “gate” that the Atlantic bluefin tuna must cross prior to reaching the GSL foraging ground. The completion of the OTN lines enabled us to record long-term movements of bluefin tuna acoustically tagged on their GSL foraging grounds. In addition, the previously deployed Halifax Line, completed in 2007, provided a line of complete coverage across the Scotian Shelf (Fig. 2). Additional deployments opportunistically of receivers along the eastern seaboard of North America from Newfoundland to the Gulf of Mexico, Bahamas and in the Strait of Gibraltar provided opportunistic detections (Fig. 3a).

State-space models offer a flexible and integrated framework for model fitting when data contain noise in addition to information about the demographic process of interest. Multistate mark–recapture models^36,37^ are a natural generalization of the Cormack-Jolly-Seber model^38^, where individuals can move between states (e.g. geographic sites or reproductive status) according to transition probabilities. Use of a Bayesian approach allows incorporation of prior knowledge from other studies or sources that is particularly advantageous in data-limited situations. We developed a Bayesian state space formulation of the multistate mark recapture model^39^ for acoustic tagged Atlantic bluefin tuna, in which states correspond to geographic areas and whether an individual carries a functioning or non functioning acoustic tag. The multistate state-space Bayesian mark-recapture model used to estimate survival is fully described in the Supplementary Material.

## Results

128 Vemco tags were deployed on Atlantic bluefin tuna from October 2009 to October 2016. Of these tagged fish, 124 were released in the Gulf of St. Lawrence, Canada and 4 were released off of North Carolina, USA in May 2013. The acoustic tagged Atlantic bluefin tuna ranged in measured curved fork length from 174 to 313 cm CFL, with a mean length of 248 cm (±29 cm SD) (Table 1). 91% of the acoustically tagged fish were subsequently detected by a receiver post deployment (Table 1). From these acoustic tag deployments, 31,822 acoustic detections were acquired by receivers located along the eastern seaboard of North America from Newfoundland to the Florida keys, the Bahamas, and in the Strait of Gibraltar (Figs 1–3). We used 101 acoustic tags for development of a bluefin tuna mortality model and the mean mean curved fork length for tagged fish in the model was 250 cm (5^th^ percentile 193 cm, 95^th^ percentile 294 cm).

**Figure 4:**
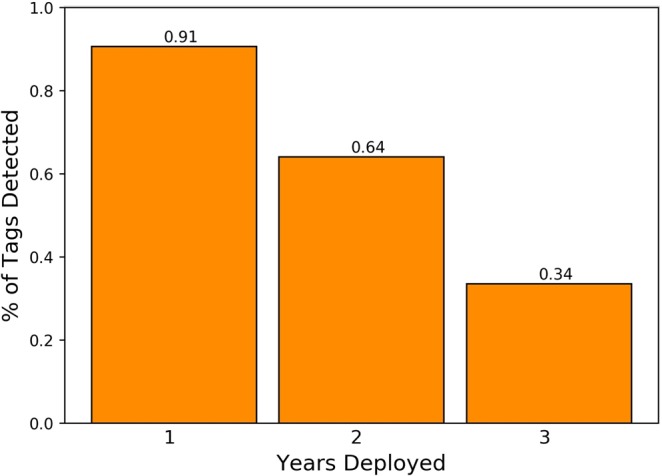
Percentage of tags detected by year. Tags were initially programmed to last 2.5 years which accounts for the drop off in detection in year 3.

The original deployment years (2009–2013) were designed to test whether the Vemco acoustic tags (V16–4h, 6 L) were detectable from bluefin of the size class tagged, and we scheduled these tags to transmit coded acoustic pulses for a period of ~2.5 years with a predicted maximum of 858 days. The tags were designed with a kill switch for 865 days per manufacturer specifications, however some variation occurs due to battery life and temperature. Up to one year post release, 91% of the acoustic tags were detected at the OTN lines in Canadian maritime Shelf waters (Fig. 4). By year two, 61% of the fish carrying acoustic tags were detected across the OTN lines. As many as 34% of the tags were still detected in their third year post release indicating that the battery life extended beyond the manufacturer specifications (Fig. 4). Two bluefin tuna tags were detected for four years post release from this first release of Vemco tagged bluefin tuna and a single fish had five years of detections also indicative that tag attachments worked.

Based on results from pop up satellite archival tagging^33^, and the recapture history of tagged bluefin tuna in the Mediterraenan Sea (Table 2), some emigration of tagged bluefin tuna out of the detection region in and around the GSL occurs each year due to trans-oceanic movements of fish to the eastern Atlantic Ocean and the Mediterranean Sea. A single fish (5111037) was detected in the Strait of Gibraltar on 26 May 2012 confirming a proportion of the population tagged in this region moves to the Mediterranean Sea post-tagging, consistent with recent satellite tag results. Four fish were recaptured in the Mediterranean Sea, one after five years post release with the acoustic tag externally intact on the fish.

**Table 2:** Recovered acoustic tags by vessel type and location.

Event ID	Tag Number	Conventional Tag	Tag Recovery	Vessel Type	Location
511009100	1101608	AY02821 AY02883	1	Recreational	North Carolina
511102000	1117034	AY02813	1	Longline	Bahamas
511104000	1101607	AY02504	1	Farm Pen	Malta
511204601	1117046		1	Commercial	Prince Edward Island
511301100	1162685	BYP021456	1	Purse Seine	Turkey
511302101	1169685	AY02607	1	Research	Gulf of St Lawrence
511302701	1169686	AY02793	0	Commercial	Prince Edward Island
511401401	1162681	AY02798	1	Commercial	Ionian Sea
511401700	1162693	AY02312	1	Purse Seine	Malta
511602001	1207120	AY03061	1	unknown	Gulf of St Lawrence

### Canadian Receivers

**Figure 5:**
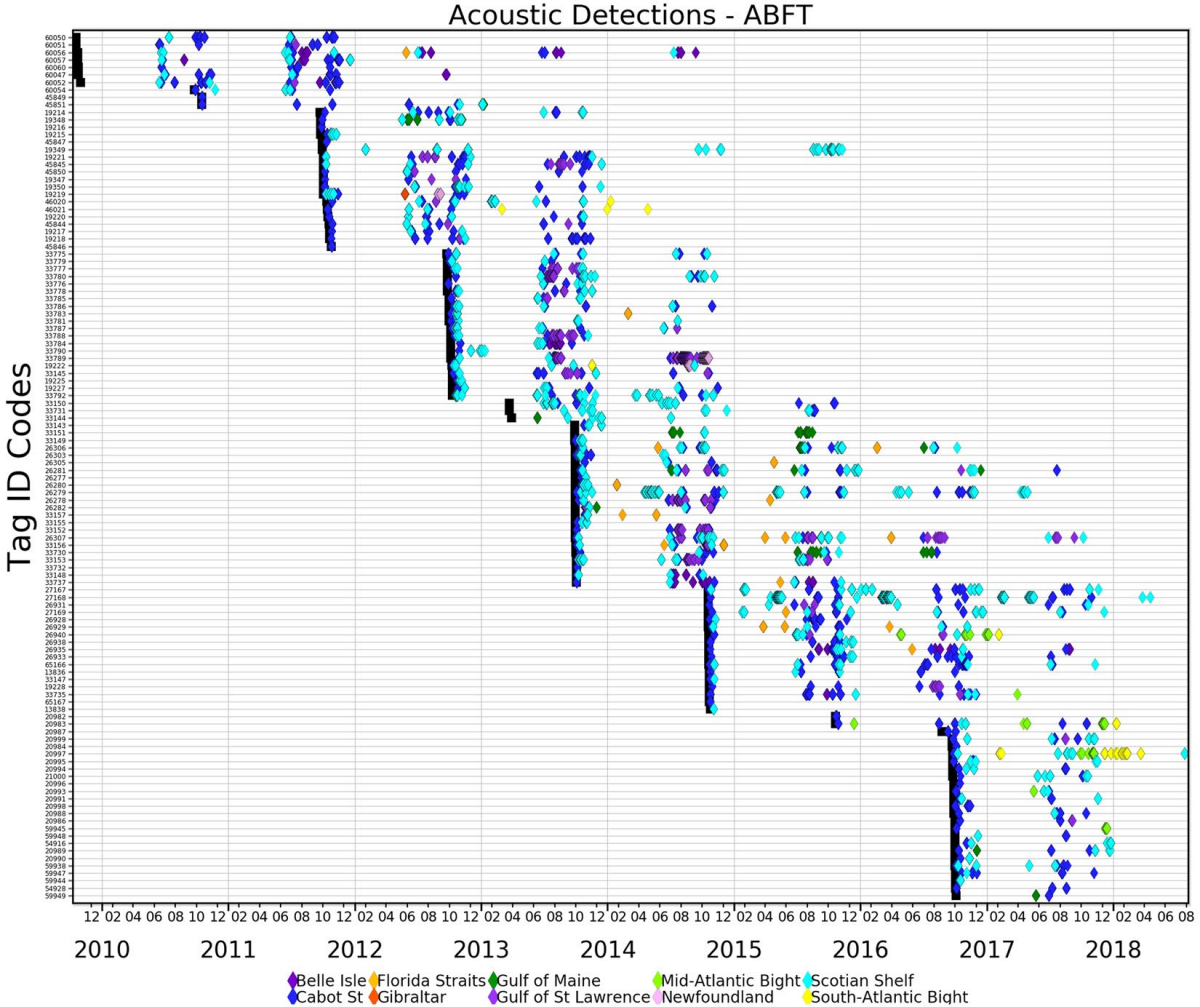
Detections of individual bluefin tuna with an acoustic tag. Eight consecutive years of deployments (black square) and subsequent acoustic detections for a fish from 2009–2016 deployments (diamonds are receiver detections colored by regions as indicated in the legend).

Most of the acoustic detections (69% or 21,816 detections) were from lines of OTN receivers located in the Cabot Strait (9082 detections of 112 individuals), the Strait of Belle Isle (605 detections of 10 individuals) and off Halifax, Nova Scotia (12,129 detections of 90 individuals) (Fig. 3b). Based on acoustic detections bluefin tuna entered the GSL by crossing the Cabot Strait Line during the summer months from 4 June to 22 October, (mean date 10 July) (Fig. 5). Bluefin exited the GSL when crossing the Cabot Strait Line from 2 July to 19 November (mean date 12 October) after spending 7 to 166 days (mean GSL residency 94 days) on the GSL foraging grounds. Bluefin tuna usually crossed the Halifax Line on the Scotian Shelf (located ~400 km southwest of the Cabot Strait Line) before crossing the Cabot Straight Line in the early summer and after in the fall. Bluefin tuna crossed the Strait of Belle Isle Line, located to the north of Newfoundland, from 7 July to 23 September (mean date 6 August), including one fish that crossed the Strait of Belle Isle Line in four consecutive years. It appears these fish were exiting the GSL via this route as most had been detected earlier entering the GSL via the Cabot Strait. Entry and exit dates, and residency days were calculated from detections post deployment year.

**Figure 6:**
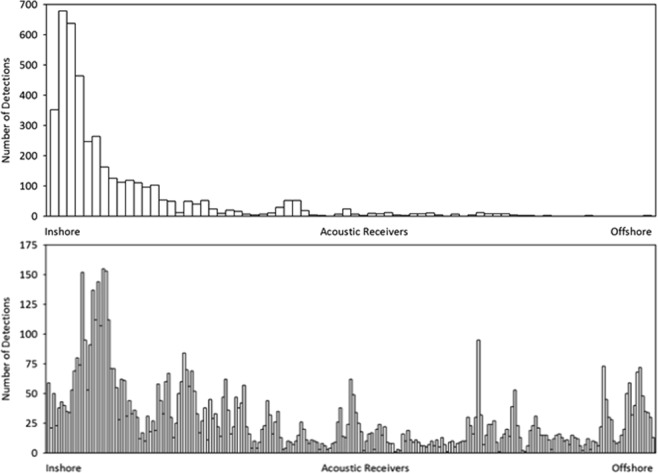
Number of acoustic detections on receivers along the Cabot Strait Line (top) and Halifax Line (bottom). Each bar represents an individual receiver.

Transit durations between the crossing of the Halifax Line on the Scotian shelf on the northern journey, and the Cabot Strait Lines ranged from 2.85 to 77 days (mean duration 14.90 days). The shortest distance that a fish could swim between the two lines is approximately 460 km, suggesting a minimum sustained speed of approximately 6.73 km/hour, in the case of the bluefin tuna with the shortest duration between subsequent recordings. Transit durations between the Cabot Strait and Halifax Lines were longer, ranging from 3.57 to 127 days (mean duration 37.53 days). Inshore receivers on both the Cabot and Halifax lines received significantly more hits than offshore receivers (Fig. 6) indicative that the fish are moving along the coastal shelf waters in relatively shallow depths.

Additional detections of tagged Atlantic bluefin in Canadian waters were obtained from Vemco Mobile Transceivers (VMTs) attached to free swimming grey seals located in the southern GSL and on the Scotian Shelf (Lidgard *et al*. 2014) and acoustic receivers located in the southern GSL (Canso Causeway, Chaleur Bay), on the Atlantic coast of Nova Scotia (St. Margaret’s Bay, Sable Island) and off Newfoundland (Fortune Bay, Twillingate). Some of these receivers provided large numbers of detections, particulary the VMTs attached to gray seals (2507 detections of 34 individuals – July to December) and the Canso Causeway (4636 detections of 6 individuals – July to October), Fortune Bay (1723 detections of 2 individuals – August to October) and Chaleur Bay receivers (1908 detections of 11 individuals – July to October).

An interesting finding of the current study was the large number of detections that we observed on the Canso Causeway receiver (detections). The Strait of Canso, linking the GSL to the Atlantic Ocean was the historic migration route of these fish, and has been blocked by the Canso Causeway since late 1952. The longevity of giant bluefin would suggest that current year classes of GSL fish are only a few generations removed from the last bluefin tuna that might have used this passage as the primary migration route for entering and exiting the southern GSL prior to 1952. There are anecdotal reports of large numbers of bluefin seen in close proximity to the causeway in the years immediately following its construction. The receiver in the vicinity of the Canso causeway obtained over 4000 detections. To exit the GSL, bluefin must swim around Cape Breton Island to reach the Atlantic side of the Strait of Canso, a detour >450 km or longer or go north thru Bell Isle.

**Figure 7:**
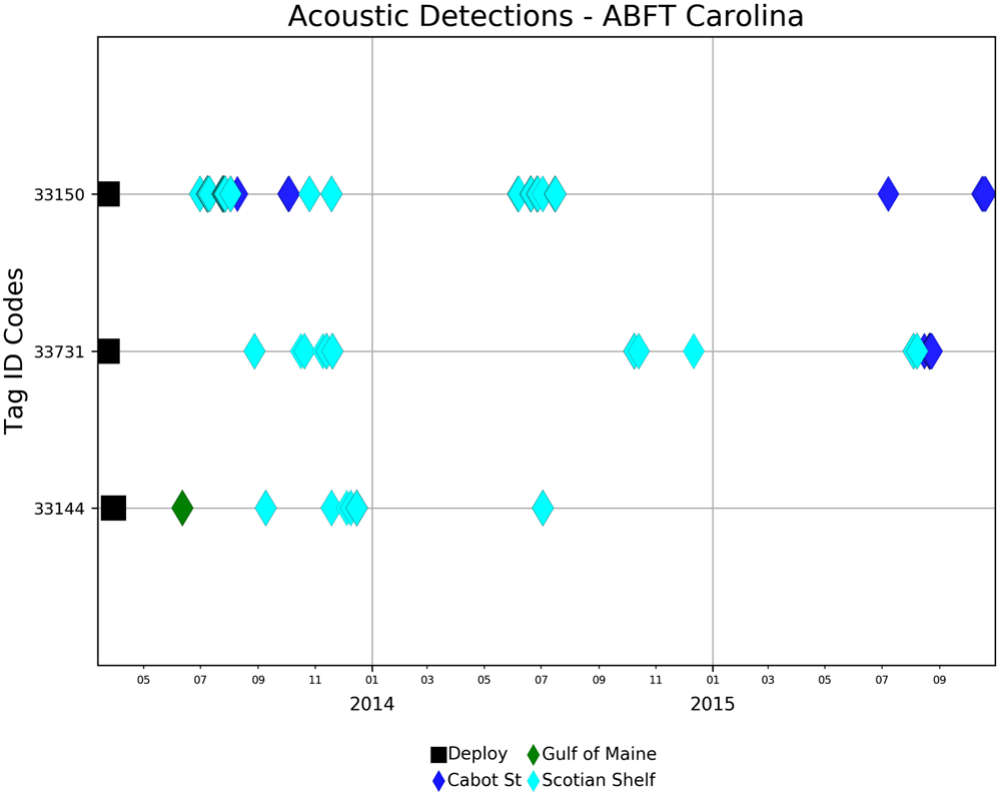
Deployents (black square) and acoustic detections (colored diamonds) of Atlantic bluefin tuna released in March, 2013 off North Carolina.

Tagged Atlantic bluefin tuna were also detected by individual moored Vemco receivers (Figs 3–5) located in the Gulf of Maine (January to May), off Cape Cod (June and November), and in the waters off Cape Hatteras (November). Additionally a few bluefin were detected in the waters off Bimini, Bahamas (January to May) and the Florida Keys (May). However, the number of detections by these receivers was small with the largest being 60 detections by GoMOOS receivers located in the Gulf of Maine. OTN conducted tests of receivers in the Strait of Gibraltar during early 2012 and deployed a line of receivers across this passage during 2013. While testing their equipment on 26 May 2012, one bluefin tagged in GSL waters was detected 22 times by 7 different receivers. Three of the four North Carolina acoustic tags were subsequently detected by acoustic receivers (Table 1) on the Halifax line and off Sable Island (Fig. 7). One of the fish was detected off Cape Cod in June 2013.

### Bayesian mark-recapture model

**Figure 8:**
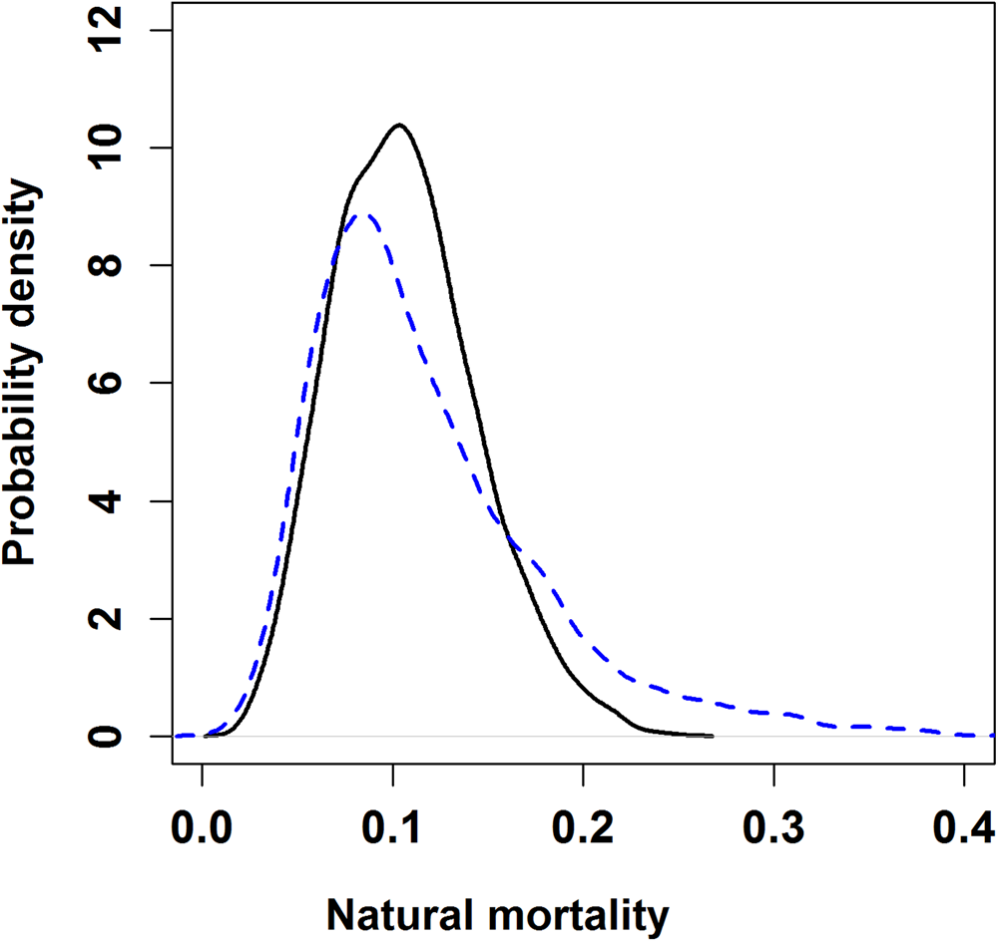
Prior (dashed blue line) and posterior pdfs for annual natural mortality.

**Figure 9:**
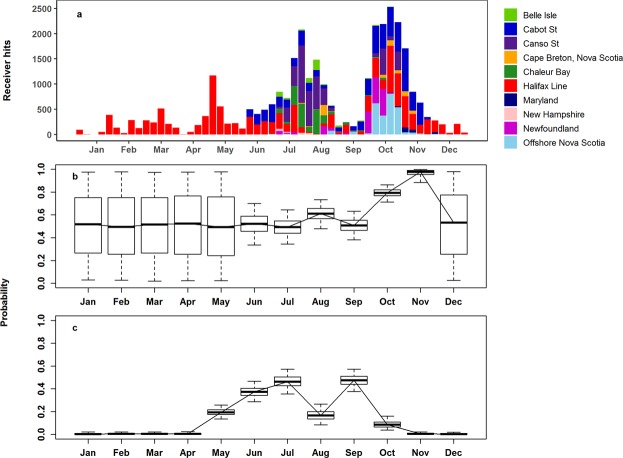
(**a**) Weekly detection frequencies by receiver array for acoustic tagged Atlantic bluefin tuna. (**b**) Posterior monthly movement rate estimates out of the Gulf of St. Lawrence (wide distributions e.g. in January and May reflect the prior). (**c**) Posterior monthly movement rate estimates into the Gulf of St. Lawrence.

Using a spatially-structured state-space model, we obtained a posterior median estimate of the instantaneous annual natural mortality rate in Atlantic bluefin tuna of 0.10 yr^−1^ (standard deviation of log *x*, SD 0.34). (Fig. 8). The acoustic tagging data were also informative about rates of seasonal movement into and out of the Gulf of St. Lawrence, upating the prior distribution in most months (Fig. 9). The estimated rate of movement into the GSLwas highest during June and September (Fig. 9b), while the high estimated rates of departure from the GSL in October and November (Fig. 9a) are consistent with observations among receivers at the Cabot, Canso and Belle Isle Straits in those months.

**Figure 10:**
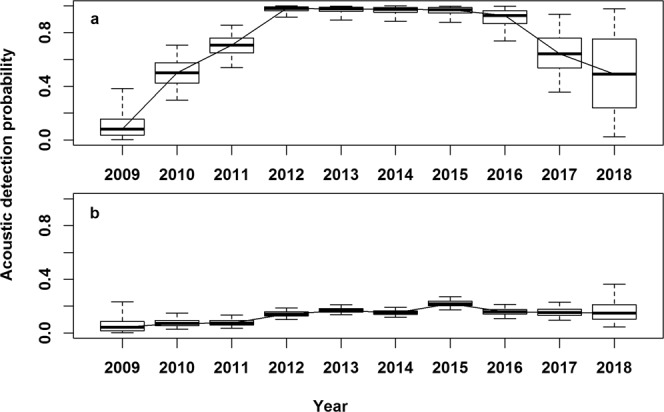
Posterior estimates of acoustic detection probabilities by year. (**a**) Gulf of St. Lawrence (**b**) outside the Gulf of St. Lawrence.

Estimated detection probabilities at acoustic receivers were much higher in the GSL box than outside (Fig. 10), reflecting a higher density of receivers in this area, and the fact that tagged Atlantic bluefin tuna must cross receiver lines to enter and exit the GSL. Acoustic detection probabilities were estimated to have increased during the first years of the study in both areas, probably reflecting recruitment of receivers in the OTN and other projects over the study’s duration. Acoustic detection probabilities in the GSL were estimated to have decreased in the final 2 years of the study (Fig. 10a), possibly reflecting attrition and re-deployments of receivers to new areas, or lags in the acquisition of receiver data annually. See supplementary material for additional model results.

## Discussion

Electronic tagging of long-lived highly migratory fishes with coded acoustic tags permits conducting long-term studies that can provide valuable information about rates of mortality and migration. For Atlantic bluefin tuna, this technology can potentially provide monitoring capacity and address significant questions such as: a) the timing of arrival and departures of Atlantic bluefin tuna foraging in Canadian waters, b) the natural mortality rate of mature fish based on Bayesian modelling approaches^17,23,35^. Acoustic tagging data can inform current population models on the status and assessment of the Atlantic bluefin tuna populations. The original battery life of the acoustic tags used in this study was programmed to be ~2.5 years. More recent tags have projected battery lives of 5–10 years. The tags showed significant reliability when placed externally, with double titanium dart attachments, indicating the technology is capable of showing fidelity to a specific geographic area. We anticipate that with the long periods of occupation evident in the GSL waters (Fig. 7b) it may be possible to routinely obtain 5 year acoustic records for Atlantic bluefin tuna. This can be utilized for long-term monitoring of the assemblage of fish in these waters and could be used to assess recruitment of juvenile fish utilizing Carolina waters into the GSL.

Atlantic bluefin tuna have a complex population structure and there remain significant questions concerning the status, the structure and dynamics of Atlantic bluefin tuna populations, especially in the North Atlantic where mixing is known to occur on foraging grounds. The availability of a network of receivers covering the Cabot Strait provided the initial opportunity to test the role of acoustic tags in improving fisheries management of these valuable fish. Development of methods to provide empirical estimates of natural mortality is of high priority for bluefin tuna stocks, since all else being equal, using a lower rate of natural mortality in the stock assessment can often lead to lower estimates of the ratio of current to unfished stock size (i.e. greater depletion), and more conservative projections of future stock development. Survival estimates from the multistate mark-recapture model for Atlantic bluefin tuna suggest a low rate of mortality from natural causes, consistent with the fact that most individuals in this study had a curved fork length ≥240  cm at tagging, corresponding to an age of ~14 years or more^39,40^. For comparison, ICCAT uses a natural mortality rate of 0.10 yr^−1^ for eastern Atlantic bluefin aged 20 years and older, and for western Atlantic bluefin tuna aged 14 and over^23^. Values used in the stock assessment are thus consistent with the natural mortality estimates obtained in this study using acoustic tag recapture histories. Acoustic tagging methods appear to have good potential to improve estimates of natural mortality in the stock assessment, where conventional tagging data have so far proven insufficient to distinguish between alternative hypotheses about natural mortality^23^.

The multistate mark-recapture model we applied provides a robust and flexible framework for estimating rates of survival and seasonal movement in long-lived migratory fish species. Disentangling non-detection, fishing vs. natural mortality and tags reaching the end of their programmed transmission life presents a challenge with acoustic tag data sets, particularly for long-lived species where relatively long recapture histories are needed to accurately estimate survivorship. Using Bayesian approaches can help to alleviate this problem by allowing incorporation of prior knowledge from other studies or sources. For example, in this study, prior information from earlier published studies was utilised for rates of natural and tagging-related mortality, while an empirical prior was developed for acoustic detection rates in the Gulf of St. Lawrence (see Supplementary Material for details). As noted above, the tags deployed from 2009–2013 had a programmed transmission life of approximately 2.5 years, which is likely not long enough to discriminate over a range of low values of natural mortality with a high degree of precision. Despite the use of prior knowledge, there is likely some conflation of natural mortality, tagging related mortality, tag loss, and non-functioning tags in model parameter estimates. Adding a further tag type to the model for which information about the reporting rate is available (e.g. tags with a large monetary reward such as the pop up satellite archival tag or surgically implanted archival tags) could help to inform estimates of acoustic tag loss and tag transmission time. The precision of the natural mortality rate estimate is also expected to improve once detection histories from tags with longer programmed transmission times (5–10 years) start to accrue.

A potential limitation of the model applied in this study is the coarse spatial resolution. Permanent (i.e. over the duration of the study) emigration out of regions of high detection probability, for example return of Mediterranean origin fish to the eastern Atlantic may affect estimates of other model parameters. This phenomenon could potentially lead to estimates of natural mortality and tag shedding rates that are biased high, although its effect is not expected to be significant given the low frequency of observations of satellite tagged Atlantic bluefin tuna that ended in the eastern Atlantic or Mediterraean (2 out of 48 over the duration of the study). Future work will extend the model to a higher spatial resolution. This could be implemented by splitting the outside-GSL box into e.g. 3 or 4 areas, allowing more detailed patterns of movement to be estimated. Improving prior information or adding auxiliary data on detection probabilities and rates of fishing mortaliy is of high priority: extension of the multistate mark-recapture model to both acoustic and satellite tag detection histories is ongoing. This is expected to improve estimates of area-specific detection probabilities and acoustic tag transmission times for acoustic tags with short detection histories. Both have potential to improve the accuracy and precision of natural mortality estimates. Given additional data on the genetic origin of tagged Atlantic bluefin tuna from fin clips (i.e. Gulf of Mexico vs. Mediterranean spawners), accounting for stock-of-origin would be straightforward within the model framework presented, whereby movement and other parameters can be estimated separately for each origin. While the results above apply to a limited number of year classes (e.g. corresponding roughly to the terminal age group in ICCAT’s western bluefin tuna assesment), there has been a trend towards smaller lengths at tagging in recent years, so that development to an age-structured model could also be of interest in future. By increasing acoustic tagging effort in North Carolina, it might also be potentially possible to determine when a fish recruits into the GSL foraging ground from this lower latitude foraging area.

Testing acoustic tagging on the GSL foraging grounds was critical as this sea is a semi-enclosed region and the OTN has strategically placed two fully closed receiver lines at Cabot Strait, and Belle Isle. This placement of receivers ensures capture of the tuna’s electronic signals when they leave the region and return. An additional line on the Scotian Shelf (Halifax Line), across the continental shelf provides valuable information in concert with the Cabot Strait line on arrival and departure. Together these receiver lines permit continuation of a long-term study both on resident and new arrivals. The GSL may serve as the best long term site for monitoring western Atlantic bluefin, due to the investment Canada has made in placing strategic underwater receiver lines here and the diligent effort they have in maintaining these lines and downloading the data. Our study has demonstrated a high detection probability within the GSL, which supports estimation of detection probabilities in other areas with lower receiver densities.

Importantly, the use of external acoustic tags was made possible only by deploying on the deck, and carefully anchoring the tag in two places. From recapture results, we know that we have succeeded in constructing a 5 year attachment tether that keeps tags on the fish reliably. Given that the V16 tags have met the 2.5 year specifications of the manufacturer in tag transmission rates we predict 5 and 10 year data detections times will be possible with the current deployment techniques (2016–present) and receiver arrays, yielding improvements in the precision of survival estimates. New models incorporating valuable information from double tag experiment (satellite and acoustic tags) data sets, as well as genetic identification of the population origin of the fish from fin clips, should improve our capacity to model the survivorship of bluefin tuna by population, providing important information on their annual foraging patterns, and potentially enabling an assessment of the efficacy of increased protections on the spawning grounds in the Gulf of Mexico.

## Supplementary information

Supplementary

## Data Availability

Telemetry data will be made available via our public website at tagging of pelagic predators (https://oceanview.pfeg.noaa.gov/topp/map) upon publication, or by request to the corresponding author. All model data is provided in the supplement.
