# Supplementary material for: Estimating Natural Mortality of Atlantic Bluefin Tuna Using Acoustic Telemetry
Source: Sci Rep. 2019 Mar 20;9:4918. doi: 10.1038/s41598-019-40065-z (PMC6426944; doi:10.1038/s41598-019-40065-z)
Supplement: Supplementary file 1 — Supplementary [file 41598_2019_40065_MOESM1_ESM.docx]

**Supplementary Information**

**Estimating Natural Mortality of Atlantic Bluefin Tuna Using Acoustic Telemetry**

Barbara A. Block*^1,2^*, Rebecca Whitlock*^2,3^*, Robbie Schallert*^2^*, Steve Wilson*^1,2^*, Michael J. W. Stokesbury*^4^* Mike Castleton*^1^*, and Andre Boustany*^5,6^*

*^1^Hopkins Marine Station, Stanford University, Pacific Grove, California 93950, USA*

*^2^Tuna Research and Conservation Center, Stanford University, Hopkins Marine Station, Pacific Grove, California, 93950, USA*

*^3^* *Swedish University of Agricultural Sciences, Department of Aquatic Resources, Institute of Freshwater Research, Stångholmsvägen 2, SE-178 93, Drottningholm, Sweden*

*^4^Biology Department, Acadia University, Wolfville, NS B4P 2R6 CANADA*

*^5^Marine Geospatial Ecology Lab, Nicholas School of the Environment, Duke*

*University, Durham NC 27708, USA*

*^6^Monterey Bay Aquarium, 886 Cannery Row, Monterey CA 93940, USA*

**Data**

Data from 101 tagged ABFT released between 2009 up to the end of 2015 are analysed in this study (Table 1). Releases from 2016 onwards were omitted, owing to truncation of the acoustic detection data for these releases (dates for the most recent receiver download available at the time of analysis ranged from November 2012 to November 2018, with a median of November 2016).

*Table S1. Summary of acoustic tag data used in the mark-recapture model.*

| ABFT ID number | Number of tags | Tagging date | CFL (cm) | First detection | Last detection | Transmission time (days) | Fishery recapture |
| --- | --- | --- | --- | --- | --- | --- | --- |
| 5109023 | 2 | 18/10/2009 | 250 | 12/07/2010 | 04/11/2011 | 815 | 0 |
| 5109024 | 2 | 18/10/2009 | 273 | 15/06/2010 | 15/09/2011 | 815 | 0 |
| 5109026 | 2 | 22/10/2009 | 269 | 20/06/2010 | 12/09/2014 | 1633 | 0 |
| 5109027 | 2 | 22/10/2009 | 293 | 22/06/2010 | 19/12/2011 | 815 | 0 |
| 5109028 | 2 | 22/10/2009 | 233 | NA | NA | 815 | 0 |
| 5109029 | 2 | 24/10/2009 | 277 | 21/06/2010 | 21/10/2011 | 815 | 0 |
| 5109030 | 2 | 24/10/2009 | 261 | 30/06/2010 | 23/09/2012 | 815 | 0 |
| 5109031 | 2 | 30/10/2009 | 268 | 15/06/2010 | 16/11/2011 | 815 | 0 |
| 5109032 | 2 | 30/10/2009 | 262 | NA | NA | 815 | 0 |
| 5110066 | 2 | 24/09/2010 | 272 | NA | NA | 815 | 0 |
| 5110067 | 2 | 24/09/2010 | 293 | 28/09/2010 | 21/10/2011 | 815 | 0 |
| 5110091 | 1 | 16/10/2010 | 197 | 17/10/2010 | 08/01/2013 | 858 | 1 |
| 5110092 | 1 | 16/10/2010 | 194 | 18/10/2010 | 18/10/2010 | 858 | 0 |
| 5111014 | 1 | 23/09/2011 | 247 | 07/10/2011 | 22/10/2013 | 858 | 0 |
| 5111018 | 1 | 24/09/2011 | 219 | 28/09/2011 | 08/11/2012 | 1633 | 0 |
| 5111019 | 1 | 24/09/2011 | 237 | NA | NA | 1633 | 0 |
| 5111020 | 1 | 24/09/2011 | 246 | 13/10/2011 | 09/11/2011 | 858 | 1 |
| 5111021 | 1 | 24/09/2011 | 299 | 27/09/2011 | 27/09/2011 | 858 | 0 |
| 5111029 | 1 | 29/09/2011 | 175 | 01/02/2012 | 09/11/2015 | 1633 | 0 |
| 5111030 | 1 | 29/09/2011 | 252 | 11/10/2011 | 11/10/2011 | 858 | 0 |
| 5111035 | 1 | 03/10/2011 | 244 | 06/10/2011 | 17/11/2012 | 1633 | 0 |
| 5111036 | 1 | 03/10/2011 | 203 | 06/10/2011 | 11/12/2013 | 1633 | 0 |
| 5111037 | 1 | 03/10/2011 | 243 | 07/10/2011 | 12/11/2012 | 858 | 0 |
| 5111038 | 1 | 03/10/2011 | 211 | 06/10/2011 | 17/11/2013 | 858 | 0 |
| 5111039 | 1 | 03/10/2011 | 239 | 06/10/2011 | 15/12/2013 | 858 | 0 |
| 5111040 | 1 | 03/10/2011 | 210 | 07/10/2011 | 19/10/2013 | 858 | 1 |
| 5111043 | 1 | 13/10/2011 | 220 | 27/10/2011 | 09/01/2014 | 1633 | 0 |
| 5111044 | 1 | 14/10/2011 | 199 | 16/10/2011 | 27/04/2014 | 1633 | 0 |
| 5111047 | 1 | 19/10/2011 | 246 | 28/10/2011 | 19/10/2013 | 858 | 0 |
| 5111048 | 1 | 21/10/2011 | 269 | 27/10/2011 | 08/11/2012 | 858 | 0 |
| 5111049 | 1 | 21/10/2011 | 209 | 25/10/2011 | 12/11/2013 | 858 | 0 |
| 5111054 | 1 | 23/10/2011 | 221 | NA | NA | 858 | 0 |
| 5111057 | 1 | 25/10/2011 | 193 | 27/10/2011 | 27/10/2011 | 858 | 0 |
| 5111058 | 1 | 16/10/2011 | 215 | 19/10/2011 | 23/10/2013 | 858 | 0 |
| 5112028 | 2 | 23/09/2012 | 270 | 27/09/2012 | 15/10/2014 | 858 | 0 |
| 5112029 | 1 | 24/09/2012 | 235 | 12/10/2012 | 21/10/2013 | 858 | 0 |
| 5112030 | 2 | 24/09/2012 | 283 | 28/09/2012 | 19/10/2013 | 858 | 0 |
| 5112031 | 1 | 24/09/2012 | 222 | 18/10/2012 | 17/11/2013 | 858 | 0 |
| 5112032 | 2 | 24/09/2012 | 260 | 01/10/2012 | 23/07/2013 | 858 | 0 |
| 5112033 | 2 | 24/09/2012 | 278 | 19/10/2012 | 05/11/2014 | 858 | 0 |
| 5112034 | 2 | 29/09/2012 | 270 | 05/10/2012 | 22/07/2014 | 858 | 0 |
| 5112035 | 2 | 29/09/2012 | 259 | 06/10/2012 | 01/03/2014 | 858 | 0 |
| 5112036 | 2 | 29/09/2012 | 261 | 06/10/2012 | 15/10/2013 | 858 | 0 |
| 5112037 | 2 | 29/09/2012 | 268 | 14/10/2012 | 30/10/2014 | 858 | 0 |
| 5112038 | 2 | 05/10/2012 | 277 | 01/11/2012 | 19/09/2013 | 858 | 0 |
| 5112039 | 2 | 05/10/2012 | 273 | 08/10/2012 | 20/07/2014 | 858 | 0 |
| 5112040 | 1 | 05/10/2012 | 218 | 15/10/2012 | 05/11/2013 | 858 | 0 |
| 5112041 | 2 | 05/10/2012 | 284 | 27/10/2012 | 20/10/2014 | 858 | 0 |
| 5112042 | 2 | 05/10/2012 | 282 | 29/10/2012 | 11/01/2013 | 858 | 0 |
| 5112043 | 1 | 08/10/2012 | 259 | 11/10/2012 | 08/09/2014 | 858 | 0 |
| 5112044 | 2 | 09/10/2012 | 265 | 24/10/2012 | 06/11/2012 | 858 | 0 |
| 5112045 | 1 | 09/10/2012 | 271 | 11/10/2012 | 21/10/2014 | 858 | 0 |
| 5112046 | 2 | 09/10/2012 | 250 | 25/10/2012 | 16/11/2014 | 858 | 1 |
| 5112047 | 1 | 09/10/2012 | 225 | NA | NA | 858 | 0 |
| 5112048 | 1 | 09/10/2012 | 221 | 18/10/2012 | 02/11/2014 | 858 | 0 |
| 5113001 | 1 | 23/03/2013 | 180 | 27/08/2013 | 23/08/2015 | 858 | 0 |
| 5113002 | 1 | 23/03/2013 | 189 | NA | NA | 858 | 0 |
| 5113003 | 1 | 23/03/2013 | 183 | 30/06/2013 | 20/10/2015 | 858 | 0 |
| 5113004 | 2 | 30/03/2013 | 174 | 11/06/2013 | 02/07/2014 | 858 | 0 |
| 5113014 | 2 | 28/09/2013 | 272 | 24/10/2013 | 14/12/2013 | 858 | 0 |
| 5113015 | 2 | 28/09/2013 | 284 | 30/09/2013 | 21/10/2013 | 858 | 0 |
| 5113016 | 2 | 28/09/2013 | 251 | 05/07/2014 | 15/08/2015 | 858 | 0 |
| 5113017 | 2 | 29/09/2013 | 282 | 03/10/2013 | 16/06/2014 | 1741 | 0 |
| 5113019 | 2 | 29/09/2013 | 262 | 26/10/2013 | 26/04/2015 | 1741 | 0 |
| 5113020 | 2 | 29/09/2013 | 294 | 17/10/2013 | 30/10/2013 | 1354 | 0 |
| 5113021 | 2 | 29/09/2013 | 265 | 14/10/2013 | 22/07/2017 | 1354 | 1 |
| 5113022 | 2 | 29/09/2013 | 271 | 16/10/2013 | 07/10/2016 | 1741 | 0 |
| 5113023 | 1 | 30/09/2013 | 271 | 09/10/2013 | 15/04/2015 | 1354 | 0 |
| 5113024 | 2 | 30/09/2013 | 274 | 16/10/2013 | 28/01/2014 | 1354 | 0 |
| 5113025 | 2 | 30/09/2013 | 269 | 03/10/2013 | 06/10/2017 | 1741 | 0 |
| 5113026 | 1 | 30/09/2013 | 246 | 04/10/2013 | 28/04/2017 | 1354 | 0 |
| 5113027 | 2 | 30/09/2013 | 296 | 28/10/2013 | 28/10/2014 | 1354 | 1 |
| 5113028 | 1 | 30/09/2013 | 239 | 04/10/2013 | 21/10/2014 | 858 | 0 |
| 5113029 | 2 | 30/09/2013 | 277 | 26/10/2013 | 22/05/2014 | 858 | 0 |
| 5113030 | 1 | 30/09/2013 | 267 | 06/10/2013 | 02/11/2013 | 858 | 0 |
| 5113031 | 2 | 01/10/2013 | 269 | 13/10/2013 | 10/08/2016 | 858 | 0 |
| 5113032 | 2 | 01/10/2013 | 313 | NA | NA | 858 | 0 |
| 5113033 | 2 | 01/10/2013 | 298 | 05/10/2013 | 02/12/2014 | 858 | 0 |
| 5113034 | 1 | 02/10/2013 | 276 | 05/10/2013 | 05/10/2013 | 858 | 0 |
| 5113035 | 2 | 02/10/2013 | 282 | 04/10/2013 | 03/10/2014 | 858 | 0 |
| 5113036 | 1 | 02/10/2013 | 241 | 05/10/2013 | 29/09/2015 | 858 | 0 |
| 5113037 | 2 | 02/10/2013 | 297 | 04/10/2013 | 06/11/2015 | 858 | 0 |
| 5114009 | 2 | 18/10/2014 | 250 | 20/10/2014 | 20/11/2017 | 1616 | 0 |
| 5114010 | 1 | 18/10/2014 | 229 | 24/10/2014 | 19/04/2018 | 1616 | 0 |
| 5114011 | 1 | 19/10/2014 | 239 | 30/10/2014 | 07/12/2017 | 1616 | 0 |
| 5114012 | 1 | 19/10/2014 | 250 | 29/10/2014 | 06/11/2016 | 1237 | 0 |
| 5114013 | 1 | 19/10/2014 | 230 | 21/10/2014 | 21/10/2017 | 1237 | 0 |
| 5114014 | 2 | 19/10/2014 | 251 | 22/10/2014 | 22/11/2015 | 1237 | 1 |
| 5114015 | 1 | 21/10/2014 | 231 | 28/10/2014 | 06/07/2017 | 1237 | 0 |
| 5114016 | 2 | 21/10/2014 | 265 | 25/10/2014 | 28/08/2017 | 1237 | 0 |
| 5114017 | 1 | 21/10/2014 | 260 | 05/11/2014 | 04/02/2017 | 1237 | 1 |
| 5114018 | 2 | 21/10/2014 | 258 | 23/10/2014 | 10/11/2017 | 2538 | 0 |
| 5114019 | 1 | 21/10/2014 | 226 | 24/10/2014 | 12/12/2015 | 1237 | 0 |
| 5114020 | 1 | 22/10/2014 | 247 | 24/10/2014 | 05/11/2014 | 858 | 0 |
| 5114021 | 1 | 22/10/2014 | 237 | 25/10/2014 | 30/03/2017 | 858 | 0 |
| 5114022 | 1 | 22/10/2014 | 252 | 29/10/2014 | 12/10/2016 | 858 | 0 |
| 5114023 | 2 | 22/10/2014 | 272 | 24/10/2014 | 24/10/2014 | 2538 | 0 |
| 5114024 | 2 | 22/10/2014 | 270 | 25/10/2014 | 30/09/2016 | 820 | 0 |
| 5114026 | 1 | 26/10/2014 | 226 | 04/11/2014 | 04/11/2014 | 820 | 0 |
| 5115001 | 2 | 22/10/2015 | 250 | 23/10/2015 | 24/10/2015 | 2538 | 0 |
| 5115002 | 1 | 22/10/2015 | 229 | 29/10/2015 | 11/01/2018 | 2538 | 0 |

**Multistate mark–recapture model**

A spatially-structured model is applied to the ABFT with areas defined as inside (area 1) and outside (area 2) the Gulf of St. Lawrence, where acoustic detection effort is concentrated (Figure S1).


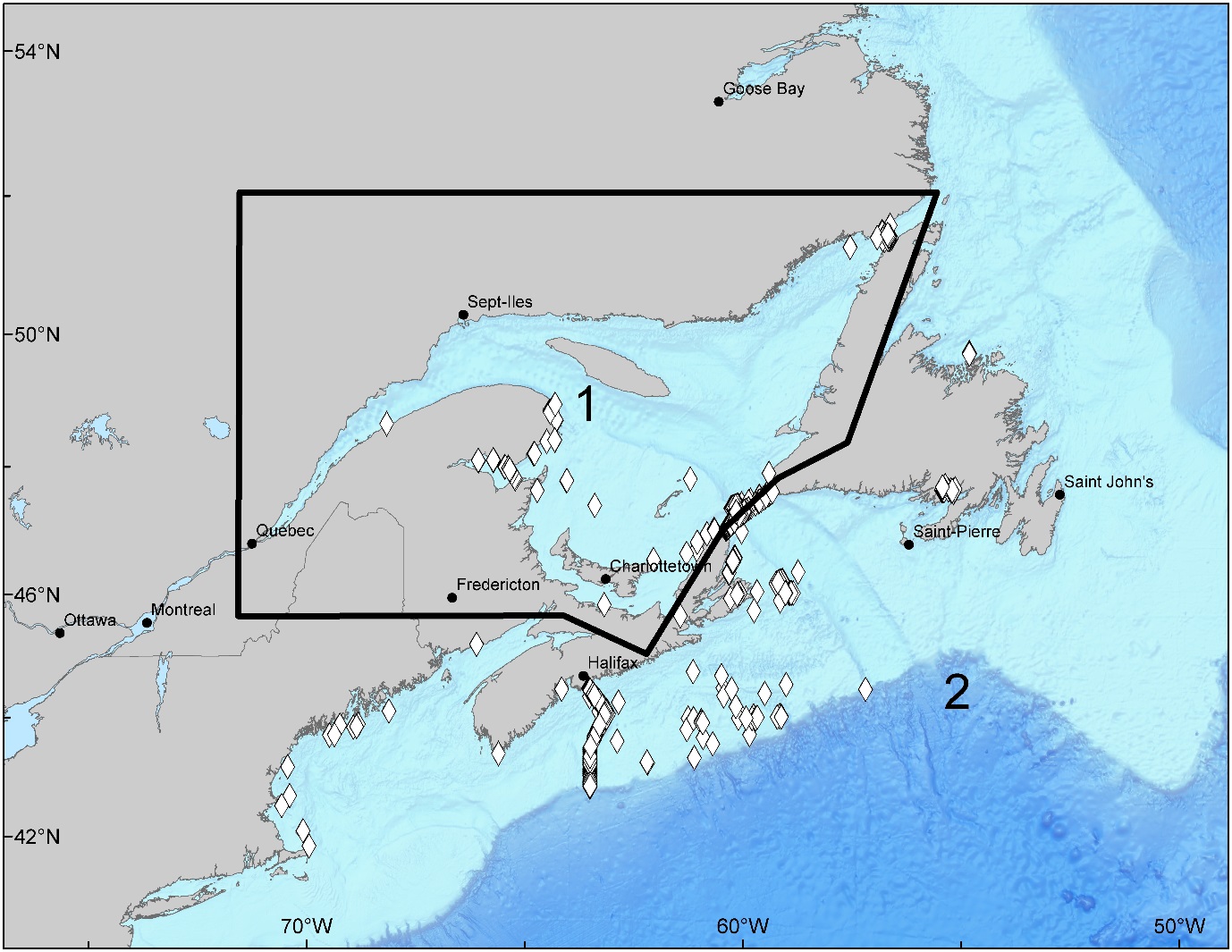
*Figure S1. Spatial areas for the multistate mark-recapture model. Area 1, inside the Gulf of St. Lawrence. Area 2, outside the Gulf of St. Lawrence. White diamonds show the positions of acoustic receivers that detected bluefin tuna. This map was generated in ESRI ArcMap software (Version:10.3.1 &* [*http://desktop.arcgis.com/en/arcmap/10.3/main/get-started/whats-new-in-arcgis-1031.htm*](http://desktop.arcgis.com/en/arcmap/10.3/main/get-started/whats-new-in-arcgis-1031.htm)*).*

State and observation vectors for acoustic tagged ABFT are defined in Table S2; parameter definitions and priors can be found in Table S3. In order to formulate a prior for acoustic tag detection probabilities in model area 1 (GSL), we used location estimates for satellite-tagged ABFT from a state-space Bayesian model^1^. The number of crossings of the Cabot straight was recorded for each satellite-tagged fish and compared to the number of crossing events that were detected on the acoustic array. Detection probabilities priors were formulated for 2 time periods (2009-2011 and 2012-2015) owing to the high rate of recruitment of receivers in the early years of the study.

*Table S2. State and observation vectors in the multistate mark–recapture model for acoustic tagged ABFT.*

| **Random state vector** $\boldsymbol{X}_{\boldsymbol{i,t}}$ | **Interpretation** |
| --- | --- |
| (1,0,0,0,0,0,0) | Alive with attached and functioning acoustic tag in area 1 |
| (0,1,0,0,0,0,0) | Alive with attached and functioning acoustic tag in area 2 |
| (0,0,1,0,0,0,0) | Alive with attached and non-functioning acoustic tag in area 1 |
| (0,0,0,1,0,0,0) | Alive with attached and non-functioning acoustic tag in area 2 |
| (0,0,0,0,1,0,0) | Dead (fishing mortality) area 1, tag attached |
| (0,0,0,0,0,1,0) | Dead (fishing mortality) area 2, tag attached |
| (0,0,0,0,0,0,1) | Dead (natural mortality) either area; alive and tag shed in either area; dead (fishing mortality) and tag shed in either area |
| **Random observation vector** $\boldsymbol{Y}_{\boldsymbol{i}\boldsymbol{,}\boldsymbol{t}}$ | **Interpretation** |
| (1,0,0,0,0) | Acoustic detection in area 1 |
| (0,1,0,0,0) | Acoustic detection in area 2 |
| (0,0,1,0,0) | Reported recapture in area 1 |
| (0,0,0,1,0) | Reported recapture in area 2 |
| (0,0,0,0,1) | Not encountered |

We used JAGS to fit the multistate model to individual tuna recapture (acoustic detection histories) via MCMC. Two MCMC chains were run, each 90 000 iterations long: a sample of 1 000 iterations from the joint posterior probability distribution was obtained by discarding the first 70 000 iterations and retaining every 20th remaining iteration thereafter. Convergence of MCMC chains to the posterior distribution was checked using the Gelman-Rubin diagnostic and other standard diagnostic plots (R package *coda*^2^).

The state-space formulation for the multistate Arnason-Schwarz model^3^ for acoustic-tagged ABFT is given by:

*State-equation*

$$X_{i,t+1}|X_{i,t}\sim Multinomial\left( 1, X_{i,t}\left[ \begin{matrix} (1-\theta_{1,s\left( t \right)})\varphi_{t,1}\rho_{t}(1-\omega_{i,t-r\left[ i \right]+1}) & \theta_{1,s(t)}\varphi_{t,2}\rho_{t}(1-\omega_{i,t-r\left[ i \right]+1}) & (1-\theta_{1,s\left( t \right)})\varphi_{t,1}\rho_{t}\omega_{i,t-r\left[ i \right]+1} & {\theta_{1,s(t)}\varphi}_{t,2}\rho_{t}\omega_{i,t-r\left[ i \right]+1} & (1-\theta_{1,s\left( t \right)})\left( 1-\varphi_{t,1} \right)\frac{F_{y\left( t \right),1}}{Z_{y\left( t \right),1}}\rho_{t} & \theta_{1,s\left( t \right)}\left( 1-\varphi_{t,2} \right)\frac{F_{y\left( t \right),2}}{Z_{y\left( t \right),2}}\rho_{t} & *^{1} \\ \theta_{2,s(t)}\varphi_{t,1}\rho_{t}(1-\omega_{i,t-r\left[ i \right]+1}) & (1-\theta_{2,s\left( t \right)})\varphi_{t,2}\rho_{t}(1-\omega_{i,t-r\left[ i \right]+1}) & \theta_{2,s(t)}\varphi_{t,1}\rho_{t}\omega_{i,t-r\left[ i \right]+1} & (1-\theta_{2,s\left( t \right)})\varphi_{t,2}\rho_{t}\omega_{i,t-r\left[ i \right]+1} & \theta_{2,s\left( t \right)}\left( 1-\varphi_{t,1} \right)\frac{F_{y\left( t \right),1}}{Z_{y\left( t \right),1}}\rho_{t} & (1-\theta_{2,s\left( t \right)})\left( 1-\varphi_{t,2} \right)\frac{F_{y\left( t \right),2}}{Z_{y\left( t \right),2}}\rho_{t} & *^{2} \\ 0 & 0 & (1-\theta_{1,s\left( t \right)})\varphi_{t,1}\rho_{t} & \theta_{1,s(t)}\varphi_{t,2}\rho_{t} & (1-\theta_{1,s\left( t \right)})\left( 1-\varphi_{t,1} \right)\frac{F_{y\left( t \right),1}}{Z_{y\left( t \right),1}}\rho_{t} & \theta_{1,s\left( t \right)}\left( 1-\varphi_{t,2} \right)\frac{F_{y\left( t \right),2}}{Z_{y\left( t \right),2}}\rho_{t} & *^{3} \\ 0 & 0 & \theta_{2,s(t)}\varphi_{t,1}\rho_{t} & (1-\theta_{2,s\left( t \right)})\varphi_{t,2}\rho_{t} & \theta_{2,s\left( t \right)}\left( 1-\varphi_{t,1} \right)\frac{F_{y\left( t \right),1}}{Z_{y\left( t \right),1}}\rho_{t} & (1-\theta_{2,s\left( t \right)})\left( 1-\varphi_{t,2} \right)\frac{F_{y\left( t \right),2}}{Z_{y\left( t \right),2}}\rho_{t} & *^{4} \\ 0 & 0 & 0 & 0 & 0 & 0 & 1 \\ 0 & 0 & 0 & 0 & 0 & 0 & 1 \\ 0 & 0 & 0 & 0 & 0 & 0 & 1 \end{matrix} \right] \right)$$

where subscripts *i* and *t* denote individual and time step, respectively. *r[i]* denotes the time step of release for individual *i* and *s[t]* denotes the season of the year for time step *t*. In this analysis we use a monthly time step ($s=1:12).$

Terms $*^{1}$ to $*^{4}$ gather together fish that survived but shed their tag, died from natural causes, or shed their tag and were recaptured:

$$*^{1,3}=(1-\theta_{1,s\left( t \right)})\varphi_{t,1}{(1-\rho}_{t})+\theta_{1,s\left( t \right)}\varphi_{t,2}{(1-\rho}_{t})+(1-\theta_{1,s\left( t \right)})\left( 1-\varphi_{t,1} \right)\frac{M}{Z_{y\left( t \right),1}}+\theta_{1,s\left( t \right)}\left( 1-\varphi_{t,2} \right)\frac{M}{Z_{y\left( t \right),2}}+(1-\theta_{1,s\left( t \right)})\left( 1-\varphi_{t,1} \right)\frac{F_{y\left( t \right),1}}{Z_{y\left( t \right),1}}{(1-\rho}_{t})+\theta_{1,s\left( t \right)}\left( 1-\varphi_{t,2} \right)\frac{F_{y\left( t \right),2}}{Z_{y\left( t \right),2}}{(1-\rho}_{t})$$

$$*^{2,4}={\theta_{2,s\left( t \right)}\varphi_{t,1}{(1-\rho}_{t})+{(1-\theta}_{2,s\left( t \right)})\varphi_{t,2}{(1-\rho}_{t})+\theta}_{2,s(t)}\left( 1-\varphi_{t,1} \right)\frac{M}{Z_{y\left( t \right),1}}+\left( 1-\theta_{2,s\left( t \right)} \right)\left( 1-\varphi_{t,2} \right)\frac{M}{Z_{y\left( t \right),2}}+\theta_{2,s\left( t \right)}\left( 1-\varphi_{t,1} \right)\frac{F_{y\left( t \right),1}}{Z_{y\left( t \right),1}}{(1-\rho}_{t})+{(1-\theta}_{2,s\left( t \right)})\left( 1-\varphi_{t,2} \right)\frac{F_{y\left( t \right),2}}{Z_{y\left( t \right),2}}{(1-\rho}_{t})$$

$\varphi$ parameters (survival probabilities) are obtained using year- and area- specific rates of fishing mortality together with natural mortality (information about fishing mortality in the model comes from tag recaptures):

$$\varphi_{t,k}=\exp\left( -\left( M+F_{y\left( t \right),k} \right) \right)$$

where $M=M.yr/12$ and $F_{y,k}={F.yr}_{y.k}/12$. The rate of total instantaneous mortality during time step $t$ in area $k$, $Z_{y\left( t \right),k}$ is defined as:

$Z_{y\left( t \right),k}=$ $M+F_{y\left( t \right),k}$.

$\omega_{i,t}$ terms give the probability that an acoustic tag will malfunction or cease to transmit *t* time steps after release:

$\omega_{i,t}=o$, $t=1$

$\omega_{i,t}=\frac{1}{1+exp(-\upsilon_{\omega}(t-W_{i}))}$, $t=2,\ldots N$

where $o$ describes the proportion of acoustic tags that does not transmit from deployment, and $W_{i}$ is the duration of time elapsed since release at which the probability of malfunction equals 0.50 for individual *i*. $\upsilon_{\omega}$ is the slope of the logistic curve for acoustic tag life, describing the variability in time to malfunction/end of transmission. Information about priors for $W_{i}$ values (in days) can be found in Tables S1 and S3. We also performed a sensitivity analysis with:

$\omega_{i,t}=\frac{1}{1+exp(-\upsilon_{\omega}(t-W_{i}))}$, $t=1,\ldots N$

i.e. no different assumption about tag transmission for the release time step, to check the effect on survival estimates.

In the model for acoustic tags, $\rho_{t}$terms denote the product of tag retention, and the proportion of fish that survives tag-related mortality (applied to the release time step only):

$\rho_{t}=exp(-S)(1-M_{ini})$ $t=1$

$\rho_{t}=exp(-S)$  $t=2,\ldots N$

where $M_{ini}$ is the proportion of fish that dies from tagging related causes, and $S$ is the tag shedding rate, given by:

$$S=-log(1-\beta)/12$$

*Observation model*

$$Y_{i,t}|X_{i,t}\sim Multinomial\left( 1, X_{i,t}\left[ \begin{matrix} \pi_{y\left( t \right),1} & 0 & 0 & 0 & 1-\pi_{y\left( t \right),1} \\ 0 & \pi_{y\left( t \right),2} & 0 & 0 & 1-\pi_{y\left( t \right),2} \\ 0 & 0 & \lambda& 0 & 1-\lambda\\ 0 & 0 & 0 & \lambda& 1-\lambda\\ 0 & 0 & 0 & 0 & 1 \end{matrix} \right] \right)$$

*Table S3. Parameters estimated in the mark-recapture model for ABFT and their priors. Standard deviations of Lognormal distributions are given as the standard deviation of log(x).*

| Parameter | Definition | Prior |
| --- | --- | --- |
| $M.yr$ | Annual instantaneous rate of death from natural causes | Posterior mean for ages 9+^4^. Lognormal prior: mean 0.12, standard deviation 0.50. |
| $\pi_{y,k}$ | Probability of detecting an acoustic tagged ABFT in year *y* on a hydrophone in area *k* | Inside GSL: Beta(1,7) prior, 2009-2011; Beta(29,1) prior, 2012-2015;  Beta(1,1) prior, 2016-2018.  Outside GSL: uninformative Beta(1,1) prior. |
| $\theta_{j,s}$ | Probability of moving out of area *j* in season *s* | Uninformative Beta(1,1) prior. |
| $\nu_{\omega}$ | Slope of the logistic curve for acoustic tag life | Lognormal: median 0.5, standard deviation 0.45. |
| $W_{i}$ | Number of time steps at which the probability that an acoustic tag stops transmitting equals 50% | Vemco tests of tag battery life, plus tests of tags with progammed kill switch. Lognormal prior: median equal to expected transmission time (see Table S1), standard deviation 0.20. |
| ${F.yr}_{y,k}$ | Instantaneous rate of death from fishing mortality in year *y* and area $k$ | Lognormal; mean 0.10, standard deviation 0.71. |
| $\lambda$ | Probability that a recaptured tag is reported | Uninformative Beta(1,1) prior |
| $\beta$ | Annual proportion of tags that is shed | Informative Beta(1,19) prior^4^ |
| $o$ | Proportion of tags that does not transmit from release | Beta(2.5,47.5) prior with mean 0.05. |
| $M_{ini}$ | Proportion of tagging related mortalities | Based on estimate of catch and release mortality for ABFT of 0.05^5^. Beta(2.5,47.5) prior with mean 0.05. |

*Table S4. Summary statistics of posterior distributions for selected model parameters; median and standard deviation. Standard deviations (SD) of Lognormal distributions are given as the SD of* $\log\left( x \right).$

| **Model parameter** | **Posterior** |
| --- | --- |
| ${F.yr}_{2009,1}$ | 0.08 (0.69) |
| ${F.yr}_{2010,1}$ | 0.08 (0.65) |
| ${F.yr}_{2011,1}$ | 0.07 (0.66) |
| ${F.yr}_{2012,1}$ | 0.07 (0.60) |
| ${F.yr}_{2013,1}$ | 0.07 (0.62) |
| ${F.yr}_{2014,1}$ | 0.08 (0.70) |
| ${F.yr}_{2015,1}$ | 0.07 (0.65) |
| ${F.yr}_{2016,1}$ | 0.11 (0.61) |
| ${F.yr}_{2017,1}$ | 0.12 (0.64) |
| ${F.yr}_{2018,1}$ | 0.08 (0.70) |
| ${F.yr}_{2009,2}$ | 0.08 (0.68) |
| ${F.yr}_{2010,2}$ | 0.07 (0.64) |
| ${F.yr}_{2011,2}$ | 0.11 (0.58) |
| ${F.yr}_{2012,2}$ | 0.06 (0.57) |
| ${F.yr}_{2013,2}$ | 0.06 (0.59) |
| ${F.yr}_{2014,2}$ | 0.09 (0.63) |
| ${F.yr}_{2015,2}$ | 0.07 (0.55) |
| ${F.yr}_{2016,2}$ | 0.07 (0.53) |
| ${F.yr}_{2017,2}$ | 0.12 (0.57) |
| ${F.yr}_{2018,2}$ | 0.08 (0.68) |
| $M.yr$ | 0.10 (0.34) |
| $M_{ini}$ | 0.03 (0.02) |
| $\beta$ | 0.04 (0.03) |
| $\lambda$ | 0.28 (0.13) |
| $\pi_{2009,1}$ | 0.08 (0.10) |
| $\pi_{2010,1}$ | 0.50 (0.11) |
| $\pi_{2011,1}$ | 0.71 (0.08) |
| $\pi_{2012,1}$ | 0.98 (0.02) |
| $\pi_{2013,1}$ | 0.98 (0.03) |
| $\pi_{2014,1}$ | 0.97 (0.03) |
| $\pi_{2015,1}$ | 0.97 (0.03) |
| $\pi_{2016,1}$ | 0.93 (0.07) |
| $\pi_{2017,1}$ | 0.64 (0.15) |
| $\pi_{2018,1}$ | 0.49 (0.29) |
| $\pi_{2009,2}$ | 0.04 (0.06) |
| $\pi_{2010,2}$ | 0.07 (0.03) |
| $\pi_{2011,2}$ | 0.08 (0.02) |
| $\pi_{2012,2}$ | 0.14 (0.02) |
| $\pi_{2013,2}$ | 0.17 (0.02) |
| $\pi_{2014,2}$ | 0.15 (0.02) |
| $\pi_{2015,2}$ | 0.22 (0.03) |
| $\pi_{2016,2}$ | 0.16 (0.03) |
| $\pi_{2017,2}$ | 0.15 (0.03) |
| $\pi_{2018,2}$ | 0.15 (0.08) |

The sensitivity run with no additional proportion of tags that do not transmit from the release time step yielded a posterior median estimate of M yr^-1^ of 0.11, with a slightly higher rate of tagging induced mortality (posterior median 0.05).

**References**

1. Wilson, S.G. *et al*. Tracking the fidelity of Atlantic bluefin tuna released in Canadian waters to the Gulf of Mexico spawning grounds. *Can. J. Fish. Aquatic Sciences* **72,** 1700-1717 (2015).

2. Arnason, A. N. The estimation of population size, migration rates and survival in a stratified population. *Res. Popul. Ecol.* **15,** 1–8 (1973).

3. Plummer, M., Best, N., Cowles, K., & Vines, K. CODA: Convergence Diagnosis

and Output Analysis for MCMC. R News 6, 7–11 (2006).

4. Kurota, H. *et al.* A sequential Bayesian methodology to estimate movement and exploitation rates using electronic and conventional tag data: application to Atlantic bluefin tuna (*Thunnus thynnus*). *Can. J. Fish. Aquat. Sci.* **66,** 321–342 (2009).

5. Stokesbury, M. J. W., Neilson, J. D., Susko, E. & Cooke, S. J. Estimating mortality of Atlantic bluefin tuna (*Thunnus thynnus*) in an experimental recreational catch-and-release fishery. *Biological Conservation* **144,** 2684–2691 (2011).
